# Impact of COVID-19 Pandemic on Mechanical Reperfusion in ST-Segment-Elevation Myocardial Infarction Undergoing Primary Percutaneous Coronary Intervention: A Multicenter Retrospective Study From a Non-epicenter Region

**DOI:** 10.3389/fcvm.2021.698923

**Published:** 2021-07-22

**Authors:** Qi Mao, Jianhua Zhao, Youmei Li, Li Xie, Han Xiao, Ke Wang, Youzhu Qiu, Jianfei Chen, Qiang Xu, Zhonglin Xu, Yang Yu, Ying Zhang, Qiang Li, Xiaohua Pang, Zhenggong Li, Boli Ran, Zhihui Zhang, Zhifeng Li, Chunyu Zeng, Shifei Tong, Jun Jin, Lan Huang, Xiaohui Zhao

**Affiliations:** ^1^Department of Cardiology, Institute of Cardiovascular Research, Xinqiao Hospital, Army Medical University, Chongqing, China; ^2^Department of Cardiology, People's Hospital of Banan District, Chongqing, China; ^3^Department of Cardiology, The Fifth People's Hospital, Chongqing, China; ^4^Department of Cardiology, The Ninth People's Hospital, Chongqing, China; ^5^Department of Cardiology, People's Hospital of Dianjiang District, Chongqing, China; ^6^Department of Cardiology, Emergency Medical Center, Chongqing, China; ^7^Department of Cardiovascular Medicine, People's Hospital of Nanchuan District, Chongqing, China; ^8^Department of Cardiovascular Medicine, The Three Gorges Central Hospital, Chongqing, China; ^9^Department of Cardiac Intervention Therapy, Zhongshan Hospital District, Chongqing General Hospital, University of Chinese Academy of Sciences, Chongqing, China; ^10^Department of Cardiology, The Third Hospital District, Chongqing General Hospital, University of Chinese Academy of Sciences, Chongqing, China; ^11^Department of Cardiology, Southwest Hospital, Army Medical University, Chongqing, China; ^12^Department of Cardiology, Yongchuan Hospital, Chongqing Medical University, Chongqing, China; ^13^Department of Cardiology, Daping Hospital, Army Medical University, Chongqing, China; ^14^Department of Cardiology, The Third Affiliated Hospital of Chongqing Medical University, Chongqing, China

**Keywords:** COVID-19, ST-segment-elevation myocardial infarction, primary percutaneous coronary intervention, mechanical reperfusion, non-epicenter region

## Abstract

**Objective:** The COVID-19 pandemic placed heavy burdens on emergency care and posed severe challenges to ST-segment-elevation myocardial infarction (STEMI) treatment. This study aimed to investigate the impact of COVID-19 pandemic on mechanical reperfusion characteristics in STEMI undergoing primary percutaneous coronary intervention (PPCI) in a non-epicenter region.

**Methods:** STEMI cases undergoing PPCI from January 23 to March 29 between 2019 and 2020 were retrospectively compared. PPCI parameters mainly included total ischemic time (TIT), the period from symptom onset to first medical contact (S-to-FMC), the period from FMC to wire (FMC-to-W) and the period from door to wire (D-to-W). Furthermore, the association of COVID-19 pandemic with delayed PPCI risk was further analyzed.

**Results:** A total of 14 PPCI centers were included, with 100 and 220 STEMI cases undergoing PPCI in 2020 and 2019, respectively. As compared to 2019, significant prolongations occurred in reperfusion procedures (*P* < 0.001) including TIT (420 vs. 264 min), S-to-FMC (5 vs. 3 h), FMC-to-W (113 vs. 95 min) and D-to-W (83 vs. 65 min). Consistently, delayed reperfusion surged including TIT ≥ 12 h (22.0 vs.3.6%), FMC-to-W ≥ 120 min (34.0 vs. 6.8%) and D-to-W ≥ 90 min (19.0 vs. 4.1%). During the pandemic, the patients with FMC-to-W ≥ 120 min had longer durations in FMC to ECG completed (6 vs. 5 min, *P* = 0.007), FMC to DAPT (24 vs. 21 min, *P* = 0.001), catheter arrival to wire (54 vs. 43 min, *P* < 0.001) and D-to-W (91 vs. 78 min, *P* < 0.001). The pandemic was significantly associated with high risk of delayed PPCI (OR = 7.040, 95% CI 3.610–13.729, *P* < 0.001).

**Conclusions:** Even in a non-epicenter region, the risk of delayed STEMI reperfusion significantly increased due to cumulative impact of multiple procedures prolongation.

## Introduction

ST-segment-elevation myocardial infarction (STEMI) is a major cardiovascular emergency requiring early diagnosis and timely reperfusion (1). Mechanical reperfusion is mainly based on rapid and standardized emergency procedures for chest pain (2). Since the outbreak in December 2019, over 110 million coronavirus-2019 disease (COVID-19) infected cases have been diagnosed and 2.4 million confirmed deaths (3). The continuing pandemic placed heavy burdens on emergency care and posed severe challenges to STEMI treatment (4).

On the one hand, protective measures against COVID-19 cause delays in primary percutaneous coronary intervention (PPCI) and prolonged ischemia time thus may lead to poor prognosis. On the other hand, emergency process without protection greatly increases the risk of virus spread, especially serious infection in hospital (5, 6). Therefore, how to balance prevention and treatment is a great ordeal for medical institutions. Considering the pandemic may last for a long time, as a core issue in health governance, it will profoundly affect the public health system and chest pain practice. In previous studies, decline of admitted STEMI was reported both in Europe, US etc, and increased delays in PPCI were also observed in COVID-19 epicenters (7–9). However, in non-epicenters, few studies on detailed mechanical reperfusion characteristics were reported.

## Methods

### Study Population

This multicenter retrospective study included 14 PPCI centers, which were certified by the China Chest Pain Center (CCPC) with standardized catheterization lab. In light of changes in epidemic and adjustments in public health response, the COVID-19 pandemic was defined as the period from January 23 (the day on which Wuhan City entered into a state of full-scale wartime through the lockdown, and then other regions including Chongqing City also upgraded their public health response to prevent the spread of the epidemic) to March 29 in 2020 (the day on which Chongqing City downgraded local public health response due to the absolute clearance of COVID-19 cases). Also, similar patients at the same period last year were included to reduce the biases of seasonal variation and festive events on the incidence. The patients with confirmed or suspected COVID-19 were excluded. Our study protocol complied with the Declaration of Helsinki and was approved by Xinqiao Hospital Ethics Committee, Army Medical University.

### Treatment Procedure During the Pandemic

**Figure 1:**
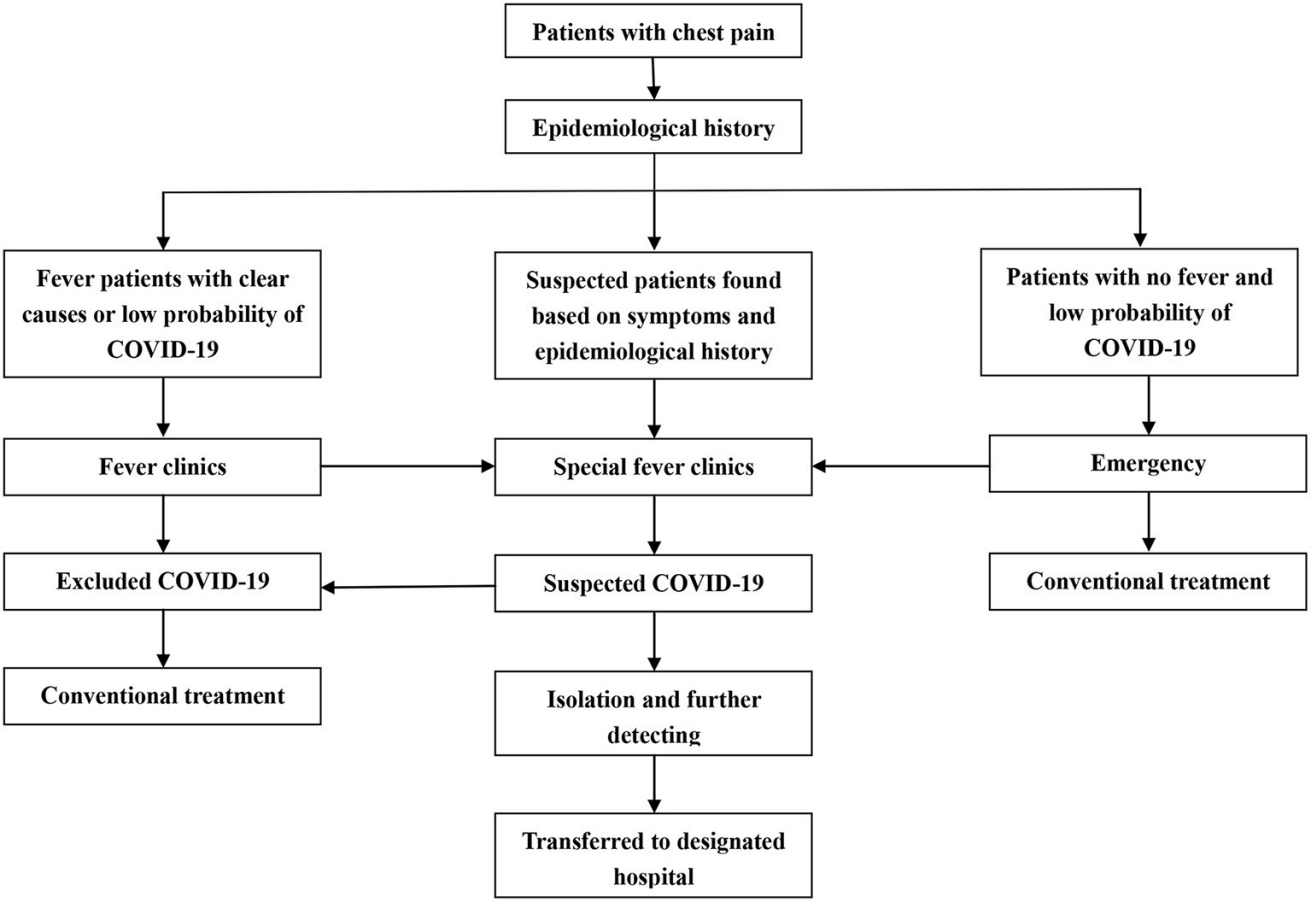
Flowchart of emergency procedure.

Although Chongqing City was a non-epicenter during the pandemic, local public health response was still upgraded on January 23 to minimize the spread of virus. Except for the lockdown, social restrictive measures were implemented to reduce external input and local transmission. For medical institutions, all admitted patients were screened for SARS-COV-2 according to Clinical Guideline of COVID-19 Diagnosis and Treatment (7 th edition) (10). In brief, the patients with confirmed or suspected COVID-19 would be transferred to the designated hospitals as soon as possible; the patients without exclusion of COVID-19 temporarily would be first transferred to the special clinics for isolation and treatment, if further tests were positive, they would be immediately transferred to the designated hospitals; while non-COVID-19 patients underwent conventional treatment procedures (11). Reperfusion therapy was determined based on benefit/risk assessment and consensus recommendation (12). Compared to the epicenters, PPCI remained the preferred option for local reperfusion therapy rather than thrombolysis-first. The flowchart of emergency procedure was shown in Figure 1.

### Definition and Data Collection

Acute myocardial infarction refers to the fourth universal definition, when troponin value exceeds the 99 th percentile upper reference limit and combines at least one of following characteristics: (1) symptoms of myocardial ischemia; (2) new changes in ischemic electrocardiogram or emerging pathological Q waves; (3) imaging evidence of new loss of viable myocardium or new regional wall motion abnormality (13). Global Registry of Acute Coronary Events (GRACE) risk score is applied to stratification and prediction of risk in patients with ACS and is calculated based on the clinical data, electrocardiogram (ECG), and laboratory parameters at admission (14).

Arrival patterns included walk-in, in-hospital onset, emergency medical services (EMS) and inter-facility transports; walk-in and in-hospital onset were defined as non-transferred pattern, while EMS and inter-facility transports were regarded as transferred pattern. PPCI parameters mainly included the period from symptom onset to first medical contact (S-to-FMC), the period from FMC to wire through culprit (FMC-to-W), and the period from door to wire through culprit (D-to-W) (15). Total ischemic time (TIT) was composed of S-to-FMC and FMC-to-W. D-to-W ≥ 90 min, FMC-to-W ≥ 120 min and TIT ≥ 12 h were deemed as pivotal timelines for delayed mechanical reperfusion (16). Clinical data and mechanical reperfusion characteristics were obtained from medical records.

### Statistical Analysis

Continuous variables are presented as mean ± SD for symmetric distributions and median (interquartile range, IQR) for skewed distributions. Categorical variables are expressed as frequency (percentage). In comparisons between groups, the *t-*test was performed for symmetric distributed variables, and nonparametric Mann-Whitney U test was applied for skewed distributed variables. Differences in categorical variables were compared by the Chi-squared test or Fisher exact test. Taking the dichotomous delay PPCI indicators as the dependent variables, we conducted logistic regression analysis to explore the association of COVID-19 pandemic with delayed mechanical reperfusion, and sub-group analysis was utilized to further assess this correlation. Two-tailed *P*-values <0.05 were considered statistically significant. All statistical analyses were performed using SPSS software version 24.0 (SPSS, Inc, Chicago, Illinois).

## Results

### Composition and Grouping

**Figure 2:**
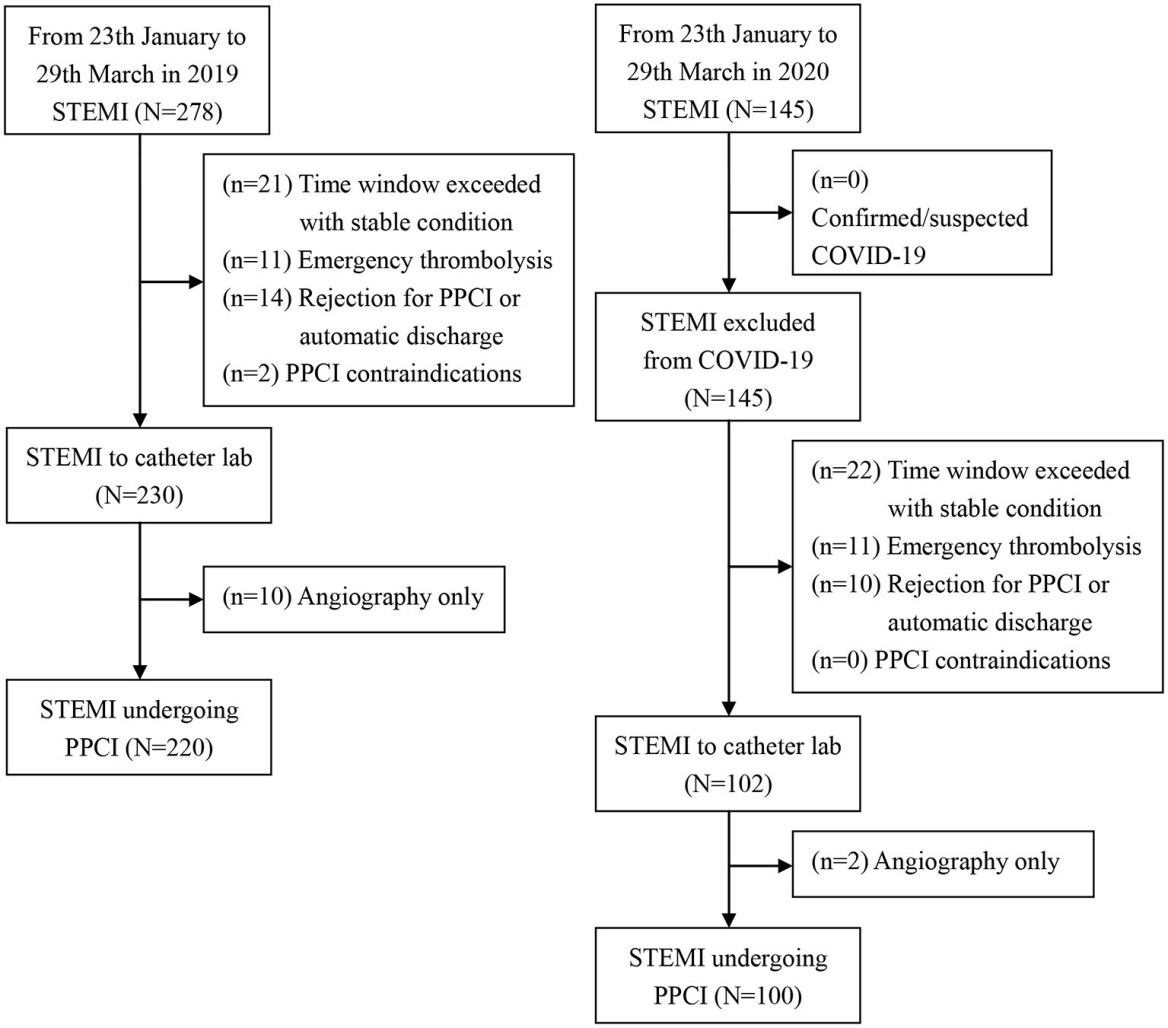
Flowchart of study population.

STEMI collaboration network from 14 PPCI centers implemented a unified procedure in accordance with CCPC specification in chest pain emergency (17). During the pandemic from 23 th January 2020 to 29 th March 2020 in China, a total of 145 consecutive patients admitted to chest pain emergency were diagnosed with STEMI, and 100 patients (69.0%) met the inclusion criteria after exclusion of non-mechanical reperfusion cases among these cases. During the same period in 2019, a total of 278 consecutive STEMI patients arrived in chest pain emergency after symptom onset, and 220 cases (79.1%) were included after screening (Figure 2).

### Comparison of Study Population Before and During the Pandemic

Overall, we identified 320 non-COVID-19 patients with STEMI undergoing PPCI as the study population (Table 1). As compared to 2019, the cases of STEMI (decreased by 47.8%) and PPCI (decreased by 54.5%) had a significant reduction. In terms of clinical characteristics, there were no differences in age, gender, heart rate, Killip class, serum creatinine and GRACE scores between the two groups (*P* > 0.05). Although arrival during non-offices hours did not differ significantly between the two groups (*P* > 0.05), more non-transferred patients with less inter-facility transports (24.0 vs. 41.4%) and more walk-in (61.0 vs. 48.6%) appeared during the pandemic (*P* < 0.05). In terms of mechanical reperfusion characteristics, significant prolongations occurred in PPCI parameters (*P* < 0.001) including TIT (420 vs. 264 min), S-to-FMC (5 vs. 3 h), FMC-to-W (113 vs. 95 min) and D-to-W (83 vs. 65 min). Further analysis revealed that median time of TIT increased by 156 min during the pandemic; COVID-19 outbreak delayed the median time of FMC-to-W for 18 min. Consistently, delayed reperfusion surged including TIT ≥ 12 h (22.0 vs.3.6%), FMC-to-W ≥ 120 min (34.0 vs. 6.8%) and D-to-W ≥ 90 min (19.0 vs. 4.1%) significantly (*P* < 0.001). Of note, the ratio of S-to-FMC to TIT increased significantly during the pandemic (72.8 vs. 63.7%, *P* < 0.001).

**Table 1:** Characteristics of study population before and during COVID-19 pandemic.

	**From 23rd January to 29th March in 2019**	**From 23 rd January to 29th March in 2020**	***P*-value**
**Characteristics**	**(*N* = 220)**	**(*N* = 100)**	
Male, *n* (%)	177 (80.5)	77 (77.0)	0.479
Age (years)	63 (54–73)	64 (55–75)	0.768
Heart rate (min)	78 (70–89)	75 (65–88)	0.082
SBP (mmHg)	122 (110–142)	125 (110–150)	0.790
DBP (mmHg)	77 (68–88)	78 (68–92)	0.667
**Killip class**			
Killip class I, *n* (%)	138 (62.7)	62 (62.0)	
Killip class II, *n* (%)	57 (25.9)	22 (22.0)	
Killip class III, *n* (%)	9 (4.1)	2 (2.0)	
Killip class IV, *n* (%)	15 (6.8)	15 (15.0)	
Killip class ≥ II, *n* (%)	82 (37.3)	39 (39.0)	0.768
Scr (μmol/L)	74.0 (61.9–90.4)	71.3 (60.3–91.4)	0.624
GRACE scores in hospital	143 (121–163)	139 (119–166)	0.804
Arrival During non-office hours, *n* (%)	99 (45.0)	51 (51.0)	0.319
**Pattern of patients arrival**			
Walk-in	107 (48.6)	61 (61.0)	
In-hospital onset	2 (0.9)	1 (1.0)	
EMS	20 (9.1)	14 (14.0)	
Inter-facility transports	91 (41.4)	24 (24.0)	
Non-transferred patients, *n* (%)	109 (49.5)	62 (62.0)	0.038
S-to-FMC (hours)	3.0 (2.0–4.5)	5.0 (3.0–10.0)	<0.001
FMC to ECG completed (min)	3 (2–6)	5 (3–7)	<0.001
Door to Troponin completed (min)	12 (11–15)	13 (12–14)	0.475
FMC to DAPT (min)	19 (17–22)	22 (19–25)	<0.001
Telephone to catheter activated (min)	9 (6–12)	13 (9–17)	<0.001
Catheter arrival to wire (min)	36 (31–42)	45 (41–53)	<0.001
D-to-W (min)	65 (57–76)	83 (75–89)	<0.001
D-to-W ≥ 90 min, *n* (%)	9 (4.1)	19 (19.0)	<0.001
FMC-to-W (min)	95 (87–108)	113 (106–124)	<0.001
FMC-to-W ≥120 min, *n* (%)	15 (6.8)	34 (34.0)	<0.001
TIT (min)	264 (204–367)	420 (295–688)	<0.001
TIT ≥ 12 h, *n* (%)	8 (3.6)	22 (22.0)	<0.001
S-to-FMC/TIT ratio (%)	63.7 (50.4–74.5)	72.8 (62.6–83.8)	<0.001
FMC-to-W/TIT ratio (%)	36.3 (25.5–49.6)	27.2 (16.2–37.4)	

*Data are expressed as median (interquartile range) or number (percentage) as appropriate*.

### PPCI Parameters Between Different Groups During the Pandemic

No differences occurred in PPCI parameters between office periods and non-office periods during the pandemic (*P* > 0.05) (Table 2). Compared to the transferred patients, the periods of FMC to ECG completed and FMC to DAPT were decreased by 2 and 3 min, respectively, in non-transferred patients (*P* = 0.002); whereas the periods of telephone to catheter activated (15 vs. 9 min, *P* < 0.001) and catheter arrival to wire (47 vs. 44 min, *P* < 0.042) significantly extended for non-transferred patients; non-transferred pattern increased the proportion of patients with TIT ≥ 12 h (*P* = 0.045) (Table 3).

**Table 2:** Comparison of PPCI parameters between different arrival periods during COVID-19 pandemic.

	**During office hours**	**During non-office hours**	***P*-value**
**Parameters**	**(*N* = 49)**	**(*N* = 51)**	
Killip class≥II, *n* (%)	18 (36.7)	21 (41.2)	0.649
GRACE scores in hospital	137 (120–161)	147 (118–172)	0.546
Non-transferred patients, *n* (%)	32 (65.3)	30 (58.8)	0.504
S-to-FMC (hours)	6.0 (3.0–11.0)	4.0 (3.0–9.0)	0.076
FMC to ECG completed (min)	5 (3–7)	6 (4–7)	0.113
Door to Troponin completed (min)	13 (11–14)	13 (12–15)	0.186
FMC to DAPT (min)	22 (19–25)	22 (19–27)	0.836
Telephone to catheter activated (min)	14 (10–17)	13 (9–18)	0.751
Catheter arrival to wire (min)	45 (41–53)	46 (42–54)	0.394
D-to-W (min)	82 (75–89)	83 (75–87)	0.753
D-to-W ≥ 90 min, *n* (%)	10 (20.4)	9 (17.6)	0.725
FMC-to-W (min)	113 (106–121)	114 (107–127)	0.574
FMC-to-W ≥ 120 min, *n* (%)	14 (28.6)	20 (39.2)	0.261
TIT (min)	462 (315–750)	374 (289–639)	0.116
TIT ≥ 12 h, *n* (%)	14 (28.6)	8 (15.7)	0.120
S-to-FMC/TIT ratio (%)	77.3 (64.3–84.0)	67.3 (58.4–82.9)	0.073
FMC-to-W/TIT ratio (%)	22.7 (16.0–35.7)	32.7 (17.1–41.6)	

*Data are expressed as median (interquartile range) or number (percentage) as appropriate*.

**Table 3:** Comparison of PPCI parameters between different transferred methods during COVID-19 pandemic.

	**Non-transferred Patients**	**Transferred Patients**	***P*-value**
**Parameters**	**(*N* = 62)**	**(*N* = 38)**	
Killip class≥II, *n* (%)	21 (33.9)	18 (47.4)	0.179
GRACE scores in hospital	134 (115–160)	143 (133–177)	0.053
Arrival during non-office hours, *n* (%)	30 (48.4)	21 (55.3)	0.504
S-to-FMC (hours)	5.5 (3.0–11.0)	5.0 (3.0–9.0)	0.322
FMC to ECG completed (min)	5 (3–6)	7 (4–10)	0.002
Door to Troponin completed (min)	13 (11–14)	14 (12–15)	0.377
FMC to DAPT (min)	21 (18–24)	24 (20–30)	0.002
Telephone to catheter activated (min)	15 (12–18)	9 (6–14)	<0.001
Catheter arrival to wire (min)	47 (42–54)	44 (36–50)	0.042
D–to-W (min)	83 (77–89)	83 (74–88)	0.511
D-to-W ≥ 90 min, *n* (%)	12 (19.4)	7 (18.4)	0.908
FMC-to-W (min)	113 (106–122)	115 (107–126)	0.649
FMC-to-W ≥ 120 min, *n* (%)	17 (27.4)	17 (44.7)	0.076
TIT (min)	443 (294–774)	400 (303–641)	0.347
TIT ≥ 12 h, *n* (%)	18 (29.0)	4 (10.5)	0.045
S-to-FMC/TIT ratio (%)	76.0 (63.1–84.5)	68.1 (62.2–82.6)	0.248
FMC-to-W/TIT ratio (%)	24.0 (15.5–36.9)	31.9 (17.4–37.8)	

*Data are expressed as median (interquartile range) or number (percentage) as appropriate*.

In Table 4, the patients with FMC-to-W ≥ 120 min had longer durations in FMC to ECG completed (6 vs. 5 min, *P* = 0.007), FMC to DAPT (24 vs. 21 min, *P* = 0.001), catheter arrival to wire (54 vs. 43 min, *P* < 0.001) and D-to-W (91 vs. 78 min, *P* < 0.001) than the patients with FMC-to-W <120 min; while S-to-FMC and TIT showed no differences between the two groups (*P* > 0.05).

**Table 4:** Comparison of parameters between timely PPCI and delayed PPCI during COVID-19 pandemic.

	**FMC-to-W <120 min**	**FMC-to-W ≥ 120 min**	***P*-value**
**Parameters**	**(*N* = 66)**	**(*N* = 34)**	
Killip class ≥ II, *n* (%)	26 (39.4)	13 (38.2)	0.910
GRACE scores in hospital	137 (115–162)	142 (123–177)	0.142
Arrival during non-office hours, *n* (%)	31 (47.0)	20 (58.8)	0.261
Non-transferred patients, *n* (%)	45 (68.2)	17 (50.0)	0.076
S-to-FMC (hours)	6.0 (3.0–9.0)	4.0 (3.0–10.0)	0.818
FMC to ECG completed (min)	5 (3–6)	6 (4–9)	0.007
Door to Troponin completed (min)	13 (11–14)	14 (12–15)	0.181
FMC to DAPT (min)	21 (18–23)	24 (20–29)	0.001
Telephone to catheter activated (min)	12 (9–16)	14 (11–20)	0.050
Catheter arrival to wire (min)	43 (40–48)	54 (47–57)	<0.001
D-to-W (min)	78 (69–83)	91 (85–106)	<0.001
D-to-W ≥ 90 min, *n* (%)	0	19 (55.9)	<0.001
FMC-to-W (min)	108 (101–113)	128 (124–138)	<0.001
TIT (min)	462 (289–654)	396 (320–724)	0.702
TIT ≥ 12 h, *n* (%)	12 (18.2)	10 (29.4)	0.199
S-to-FMC/TIT ratio (%)	77.1 (63.3–84.1)	66.3 (58.3–83.0)	0.137
FMC-to-W/TIT ratio (%)	22.9 (15.9–36.7)	33.7 (17.0–41.7)	

*Data are expressed as median (interquartile range) or number (percentage) as appropriate*.

### Association of the Pandemic With the Risk of Delayed PPCI

**Figure 3:**
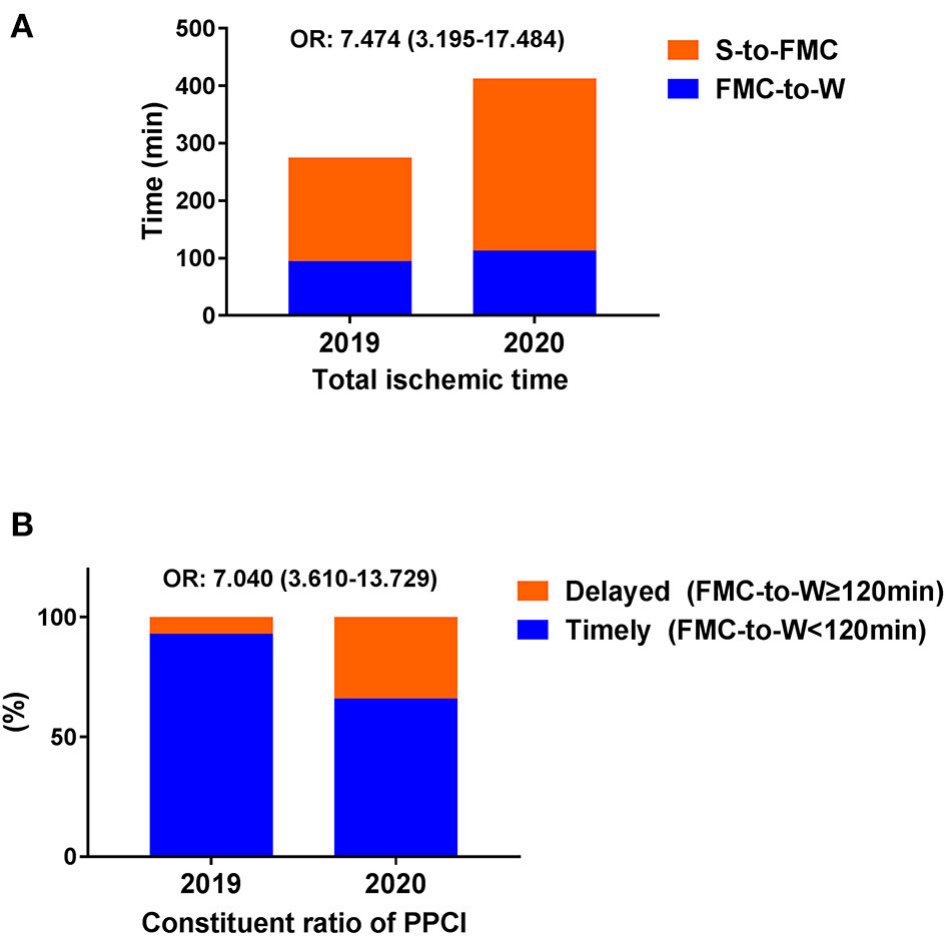
Constituent ratios of TIT and PPCI.

Logistic regression analysis was used to explore the association between the pandemic and delayed PPCI. The binary delayed PPCI indicators and COVID-19 pandemic status were included as dependent and independent variables in the model, respectively. The results indicated the pandemic was significantly associated with high risk of delayed TIT (OR = 7.474, 95% CI 3.195–17.484, *P* < 0.001), delayed FMC-to-W (OR = 7.040, 95% CI 3.610–13.729, *P* < 0.001) and delayed D-to-W (OR = 5.499, 95% CI 2.390–12.655, *P* < 0.001). Sub-group analysis stratified by clinical characteristics further examined this association (Table 5). Meanwhile, constituent ratios of TIT and PPCI were shown in Figure 3.

**Table 5:** Logistic analyses for the association of COVID-19 pandemic with delayed PPCI.

	**Delayed PPCI**								
	**TIT ≥ 12 h**			**FMC-to-W ≥ 120 min**			**D-to-W ≥ 90 min**		
	**OR**	**95% CI**	***P*-value**	**OR**	**95% CI**	***P*-value**	**OR**	**95% CI**	***P*-value**
**Overall**	7.474	(3.195–17.484)	<0.001	7.040	(3.610–13.729)	<0.001	5.499	(2.390–12.655)	<0.001
**Age**									
≥65 years	4.694	(1.733–12.717)	0.002	7.759	(3.090–19.479)	<0.001	6.803	(2.039–22.694)	0.002
<65 years	25.929	(3.189–210.805)	0.002	6.216	(2.333–16.563)	<0.001	4.353	(1.350–14.037)	0.014
**Gender**									
Male	7.475	(2.798–19.973)	<0.001	8.514	(3.849–18.832)	<0.001	5.541	(2.258–13.598)	<0.001
Female	7.235	(1.325–39.497)	0.022	4.053	(1.142–14.392)	0.030	6.300	(0.616–64.426)	0.121
**Killip class**									
Killip class ≥ II	5.850	(1.675–20.435)	0.006	6.333	(2.183–18.370)	0.001	2.868	(0.725–11.343)	0.133
Killip class < II	9.073	(2.821–29.179)	<0.001	7.525	(3.192–17.741)	<0.001	7.923	(2.707–23.190)	<0.001
**GRACE score**									
GRACE > 140	5.294	(1.930–14.524)	0.001	8.053	(3.277–19.793)	<0.001	6.568	(1.909–22.594)	0.003
GRACE ≤ 140	24.318	(3.022–195.704)	0.003	6.538	(2.367–18.062)	<0.001	4.682	(1.512–14.494)	0.007
**Office hours or not**									
Non–office hours	18.233	(2.211–150.318)	0.007	5.742	(2.425–13.598)	<0.001	3.321	(1.111–9.932)	0.032
Office hours	6.514	(2.437–17.412)	<0.001	9.280	(3.124–27.569)	<0.001	10.085	(2.641–38.518)	0.001
**Transferred or not**									
Transferred	6.412	(1.125–36.548)	0.036	9.175	(3.603–23.359)	<0.001	8.129	(1.984–33.304)	0.004
Non-transferred	7.023	(2.612–18.882)	<0.001	6.485	(2.399–17.530)	<0.001	4.120	(1.461–11.617)	0.007

## Discussion

In this study, we found that risk of delayed STEMI reperfusion significantly increased due to cumulative impact of multiple procedures in a non-epicenter region.

Evidence from Europe indicated that compared with the same period in 2019, PPCI cases decreased by 19.3%, and the median time of TIT and D-to-W were delayed by 9 and 2 min, respectively (18). The data from North America showed an estimated 38% reduction in U.S. cardiac catheterization activation after the outbreak (19). Consistently, the analysis from China's epicenter (Hubei Province) also revealed a 62.3% decline in STEMI cases during the pandemic, while the proportion of non-transferred patients characterized by walk-in increased significantly (8). In a non-epicenter, our results also revealed that a significant reduction occurred in cases of admitted STEMI and PPCI during the pandemic. Common reasons had been formulated to explain the reduction in cases including fear of infection, social distancing, and medical care avoidance. However, the decline in cases could not be simply ascribed to individual behaviors, and we should also pay attention to the comprehensive impact of the pandemic on chest pain procedure. STEMI rescue includes pre-hospital and in-hospital segments. Both S-to-FMC and D-to-W were apparently prolonged, which led to cumulative delays in reperfusion procedure.

Mechanical reperfusion for STEMI is a competition with time. The 1-year mortality of STEMI increases by 15% with every 1 h extension in time to reperfusion (20). Quality control of PPCI based on standardized procedure can help shorten TIT, reduce infarction sizes and mortality (21). Although COVID-19 has been shown to directly cause myocardial injury and induce thrombosis, heart failure, arrhythmia and even cardiac arrest; for non-COVID-19 patients, delayed PPCI affected by the pandemic might be the determinant for the poor prognosis in STEMI (22). In previous studies, Tam et al. (23) showed longer median time in all components of PPCI parameters compared with historical data from prior year in Hong Kong, yet limited by very small sample size (7 cases) and non-contemporaneous data comparison. Siudak et al. (24) reported that time from FMC to inflation significantly increased compared with analogous time period last year in Poland, but the impact of the virus infection on delayed PPCI had not been ruled out. An observational study from Canada revealed that significant delay appeared in reperfusion procedure and predominantly ascribed to patient-level and transfer-level during the pandemic (25). Of note, our study found that delays in mechanical reperfusion should be attributed to the cumulative effect of multiple processes. In addition to pre-hospital level, in-hospital delays should also not be ignored. In a non-hot spot region from America, Hammad et al. found that although no difference occurred in total D-to-B between pre-COVID-19 and post-COVID-19, a higher proportion of patients in the post-COVID-19 period presented with >12-h delay compared with the pre-COVID-19 period, and those patients with >12-h delay also had a longer average D-to-B time (26). Similarly, we also observed the adverse effect of COVID-19 pandemic on reperfusion procedure in another non-epicenter region. However, our results revealed the apparent prolongations in S-to-FMC and FMC-to-W after the outbreak through detailed parameter analysis. We speculated that this might be associated with stricter social restrictions and upgraded public health response after the first wave pandemic in China. In epicenter region (Hubei Province) from China, although differences of median time in S-to-FMC and FMC-to-W seemed to be not significant, delays in timelines was still apparent due to the highly fluctuated time and limited sample size (8). Compared with our study, reperfusion strategy of this epicenter had been adjusted to meet the needs of high-intensity epidemic control. A large number of patients from epicenter received thrombolytic therapy at the first time, given that thrombolysis could be considered as the recommended reperfusion option during the pandemic (12).

Compared to other regions, we discovered that delays in mechanical reperfusion were still rather serious in non-COVID-19 STEMI patients from a non-epicenter implying severe condition might be not the only driving factor for admission. Medical responses affected by the pandemic might be also important for seeking assistance at symptom onset. Interestingly, an observational study from Italy found that although myocardial infarction hospitalizations significantly decreased, FMC-balloon time remained unchanged after the outbreak (27). The result might be firstly attributed to the excellent reorganization for local hospital activities. Secondly, compared with our study, Italian patients were younger and had fewer cardiovascular risk factors, and were more likely to seek medical assistance timely due to striking symptoms and maintain high medical compliance in rescue procedure. FITT-STEMI study from Germany showed high-standard treatment and management for STEMI, reperfusion parameters were almost unaffected during the pandemic (16). This achievement was due to quick public response, very high proportion of EMS transport, high-level routine procedure and pre-existing care network. Based on our findings, we noticed that the pandemic might magnify the shortcomings of the pre-existing treatment pathway, thus still caused a significant delay even in a non-epicenter region. This also meant that only a high-level treatment pathway maintained for a long time could effectively deal with medical burden caused by the pandemic. In the present study, we further provided new evidence for cumulative delays in reperfusion procedure; S-to-FMC was the determinant for prolonged TIT, while slow activation in hospital was pivotal to delayed PPCI. Furthermore, our findings showed the significant correlation between the pandemic and high risk of delayed PPCI. In our opinion, longer FMC-to-W might be interpreted by institutional delays due to protective protocols for screening patients, preparing for equipment and activating personnel in catheter lab. Meanwhile, emergency care overload and staff fatigue should also be taken into consideration certainly. Hence, we proposed the insight as optimizing mechanical reperfusion by controlling cumulative delays.

## Limitations

Our study had several limitations. First, this study was subject to the biases inherent to its retrospective design. Second, clinical characteristics and PPCI parameters were evaluated by trained investigators in each center, without central reconfirmation, potentially resulting biases and errors. Third, our study had a small sample size and no follow-up data for *post-hoc* analysis.

## Conclusion

The COVID-19 pandemic significantly increased the risk of delayed STEMI reperfusion in a non-epicenter region, probably due to cumulative impact of multiple procedures prolongation.

## Data Availability Statement

The raw data supporting the conclusions of this article will be made available by the authors, without undue reservation.

## Ethics Statement

The studies involving human participants were reviewed and approved by Xinqiao Hospital Ethics Committee, Army Medical University. Written informed consent for participation was not required for this study in accordance with the national legislation and the institutional requirements.

## Author Contributions

XZ was responsible for the design. QM, JZ, JC, QX, ZX, YY, YZ, QL, XP, ZheL, BR, ZZ, ZhiL, CZ, and ST contributed to collect and clean the data. QM, JZ, YL, LX, HX, KW, and YQ performed the data analysis. QM wrote the draft of this manuscript. JJ, LH, and XZ contributed to the writing and revision of the paper. All authors contributed to the article and approved the submitted version.

## Conflict of Interest

The authors declare that the research was conducted in the absence of any commercial or financial relationships that could be construed as a potential conflict of interest.

## Abbreviations

COVID-19: coronavirus-2019 disease
STEMI: ST-segment-elevation myocardial infarction
PPCI: primary percutaneous coronary intervention
SBP: systolic blood pressure
DBP: diastolic blood pressure
Scr: serum creatinine
EMS: emergency medical service
CCPC: China Chest Pain Center
FMC: first medical contact
S-to-FMC: symptom onset to FMC
DAPT: dual antiplalet therapy
D-to-W: door to wire
FMC-to-W: FMC to wire
TIT: total ischemic time.
